# Continual Reinforcement Learning for Quadruped Robot Locomotion

**DOI:** 10.3390/e26010093

**Published:** 2024-01-22

**Authors:** Sibo Gai, Shangke Lyu, Hongyin Zhang, Donglin Wang

**Affiliations:** 1 School of Computer Science, Fudan University, Shanghai 200433, China; 17114010010@fudan.edu.cn or gaisibo@westlake.edu.cn; 2 School of Engineer, Westlake Univercity, Hangzhou 310030, China; lyushangke@westlake.edu.cn (S.L.); zhanghongyin@westlake.edu.cn (H.Z.)

**Keywords:** continual learning, quadruped robot locomotion, reinforcement learning, plasticity, entropy

## Abstract

The ability to learn continuously is crucial for a robot to achieve a high level of intelligence and autonomy. In this paper, we consider continual reinforcement learning (RL) for quadruped robots, which includes the ability to continuously learn sub-sequential tasks (plasticity) and maintain performance on previous tasks (stability). The policy obtained by the proposed method enables robots to learn multiple tasks sequentially, while overcoming both catastrophic forgetting and loss of plasticity. At the same time, it achieves the above goals with as little modification to the original RL learning process as possible. The proposed method uses the Piggyback algorithm to select protected parameters for each task, and reinitializes the unused parameters to increase plasticity. Meanwhile, we encourage the policy network exploring by encouraging the entropy of the soft network of the policy network. Our experiments show that traditional continual learning algorithms cannot perform well on robot locomotion problems, and our algorithm is more stable and less disruptive to the RL training progress. Several robot locomotion experiments validate the effectiveness of our method.

## 1. Introduction

In recent years, deep reinforcement learning (RL) has shown remarkable success in various decision-making tasks [1,2,3,4], especially in the field of robot manipulation. The success of these tasks can be attributed to a nearly stable manipulation environment (for inference), a high-quality simulation platform, and also multi-task behavioral data at this stage. Naturally, prior works thus conduct a multi-task RL formulation [5,6], exploiting the potential behavioral correlations between tasks to further boost the performance. However, the same level of success progress has not been achieved in the context of complex locomotion tasks for quadruped robots. The primary reasons behind this challenge are the lack of a stable inference environment (diverse quadruped locomotion terrains), an accurate simulator, and a publicly verifiable dataset for quadruped robots to date. Further, this makes it difficult to model the quadruped locomotion as a traditional multi-task objective. Therefore, a question naturally arises: is there any other way, besides the multi-task paradigm, for training reliable quadruped robots?

**Figure 1:**
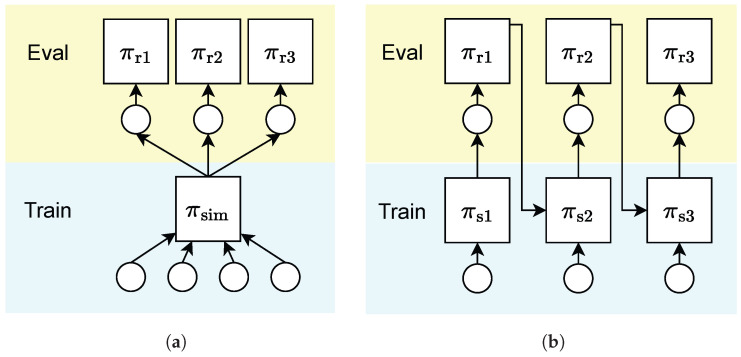
(**a**) Multi-task RL formulation: the agent learns from a set of pre-defined environments. (**b**) Continual RL formulation: the agent learns from sequential environments.

We note that even without the presence of an accurate simulator, as well as the pre-collected multi-task offline data, it is feasible to learn a base quadruped policy in a single, static environment. Therefore, we can use base policy (or other behavior policies) to collect new offline data by visiting new tasks (e.g., terrains and commands) and then further boost the policy. In this way, we can continuously learn in multiple environments, and each new policy is trained to be adaptable to the corresponding environment. As depicted above, we thus propose a continual learning approach for quadruped locomotion tasks, shifting the demanding training requirements of a multi-task formulation (see Figure 1).

The decision to model quadruped robot locomotion as a continual learning problem is driven by several key factors. Firstly, continual learning offers the advantage of adaptability and flexibility, enabling the quadruped robot to continually learn and improve its locomotion skills with new experiences. This is particularly crucial in dynamic and real-world scenarios where the quadruped environment and task requirements may change over time. Also, robot locomotion for complex tasks in large spaces is, itself, a combination of numerous tasks. New tasks will naturally emerge as the exploration capabilities of the quadruped robot improve. Secondly, continual learning allows for the accumulation of knowledge, enabling the quadruped robot to build upon its previous experiences and avoid catastrophic forgetting. This is especially important for long-term deployment and robust quadruped locomotion performance.

However, modeling quadruped robot locomotion as a continual learning problem poses several challenges that need to be addressed.

Firstly, the issue of catastrophic forgetting arises, where the robot may lose previously acquired locomotion skills when learning new tasks. Secondly, the loss of plasticity problem becomes obvious, the plasticity of the RL network will be soon saturated after learning several tasks. Additionally, the trade-off between the exploitation of existing knowledge and the exploration of new locomotion strategies needs to be carefully managed to ensure continual improvement.

To overcome these challenges, our approach incorporates a dynamic-architecture strategy. Dynamic-architecture-based algorithms allow the robot to select and learn the most critical parameters for each task, and save and lock the learned parameters for each task. On the one hand, saving and locking these parameters ensures that the robot does not forget learned skills. On the other hand, the robot can also select and leverage parameters learned in the past, allowing the robot to build on previous experience. We also indicate that re-initialization the parameters and appending the entropy of the output of the policy network can help the robot maintain its plasticity.

The main contributions of this paper are as follows:

- We introduce the concept of continual reinforcement learning for quadruped robot locomotion, addressing the limitations imposed by the lacking requirements of multi-task formulation.
- We propose a dynamic-architecture-based framework that enables continual learning, relieves the catastrophic forgetting in quadruped robot and improves complex locomotion capabilities. Then, we introduce the re-initialization and the entropy to help the robot maintain its plasticity.
- We present experimental results demonstrating the effectiveness of our approach in achieving robust and adaptive locomotion performance in dynamic environments.

In the following sections, we will discuss the challenges associated with modeling quadruped robot locomotion as a continual learning problem and present our approach to address these challenges. We will then present experimental results and discuss the implications of our findings.

## 2. Materials and Methods

### 2.1. Preliminary

In this work, we model the interaction between the quadruped robot and the environment as a partially observable Markov decision process (POMDP), denoted by the tuple $M=\{S,\Omega ,A,T,O,r,\gamma ,p_{0},\rho_{0}\}$ , where S is the full state space, Ω is the observation space, A is the action space, T:S×A×S→R is the transition dynamics function, O:S×A→Ω is the observation probability, r:S×A→R is the reward function, γ is the discount factor, $p_{0}$ is the distribution of initial states, and $\rho_{0}$ is the distribution of initial observation.

The goal in a reinforcement learning (RL) decision-making task is to learn a policy $\pi_{\theta }\left(a|o\right)$ that maximizes the expected sum of discounted rewards $E_{\pi_{\theta }\left(\tau \right)}\left[\sum_{t=0}^{T-1}\gamma^{t}r\left(s_{t},a_{t}\right)\right]$ , where $\tau :=\{s_{0},o_{0},a_{0},\cdots ,s_{K-1},o_{K_{1}},a_{K-1}\}$ denotes the trajectory and the generated trajectory distribution $\pi_{\theta }\left(\tau \right)=A_{0}\prod_{t=1}^{K-1}\pi_{\theta }\left(a_{t}|o_{t}\right)O\left(o_{t}|s_{t},a_{t-1}\right)T\left(s_{t}|s_{t-1},a_{t-1}\right)$ , where *K* is the upper limit of the number of running steps and $A_{0}=p_{0}\left(s_{0}\right)O\left(s_{0}\right)\pi_{\theta }\left(a_{0}|o_{0}\right)$ .

We would like to emphasize that our method can also be used in the Markov decision process (MDP) environment. We model the environment into a POMDP because the robot locomotion task usually model the environment into a POMDP. The propose of our continual RL method is to not affect the origin method much and keep the plug-and-play capability.

### 2.2. Continual Quadruped Robot Locomotion

Compared to other robot control tasks, quadruped robots have greater flexibility in exploring a complex real world. In the exploration process, quadruped robots will naturally encounter more environments and tasks. However, compared to the complex real world, the environments and tasks that a robot can explore in a given time are limited. Also, due to limited onboard resources, robots cannot store all the experiences they have explored. Therefore, we define the learning process of quadruped robot locomotion as a continual learning process, where the current task refers to the locomotion task within the region that a robot can freely explore when the environment, task, and physical structure of the robot remain unchanged. As soon as the environment, task, or robot dynamics change, or the physical structure that the robot can freely explore changes, the robot is faced with a new task.

We define a robot locomotion RL task as a continual RL process in which the robot has to learn a sequence of tasks $\left\{T_{n}\right\},n\in \left\{1,\cdots ,N\right\}$ , where *N* is the length of the sequence. Considering that tasks faced by robots can be repetitive, we consider each task to be sampled from a task set $T=\left\{T_{1},\cdots ,T_{\tilde{N}}\right\}$ , where $\tilde{N}$ is the set of all tasks, $\tilde{N}<N$ . In the remainder of this paper, we will use $T_{n}$ to denote the task the robot is learning, $T^{\prime }$ to denote the set of all learned tasks, and $T_{n}^{\prime }\in T^{\prime }$ to denote a previous task. For convenience, we will omit the subscript of the task index ${*}_{n}$ if we do not specify a particular task.

In the next section, we introduce the Piggyback network [7] to relieve the catastrophic forgetting problem for the Q network, the policy network, and the CENet (all three networks adopt the same anti-forgetting method).

### 2.3. Catastrophic Forgetting and Piggyback

**Figure 2:**
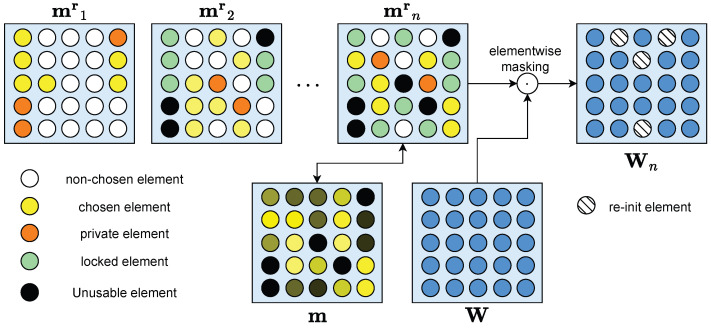
For each task, we only select the most important subset of parameters to compose a sub-network for training and utilization. Among them, only the parameters never selected by other tasks before (chosen elements) will be updated in order not to affect learned task performance, while parameters previously selected by other tasks are used without updating (locked elements). After learning each task, some parameters will be selected (private elements) for later fine-tuning on this task. Private parameters of previous tasks (unusable elements) avoid being chosen by subsequent tasks for both training and utilization thus preventing influencing the performance of other tasks. After all, those parameters that never chosen by any task will be re-initialization after training a task.

Our architecture of each network is shown in Figure 2, which is based on the Piggyback algorithm.

First, when learning the first task $T_{1}$ , for the policy network π , the critic network *Q*, and the CENet, the algorithm proposes to select and fine-tune a part of the parameters $W_{1}$ to consist a sub-network from the intact parameter W . For simplicity, we will use $W={\left[w\right]}_{ij}$ and $W_{1}={\left[w_{1}\right]}_{ij}$ if the network is not specified. In the following, we will refer to the three as the base network and the dense network, respectively. To do this, our approach learns both the parameters of the base network W and the parameters of the binary mask $m_{1}$ , and masks the base network with the binary mask $W_{1}=W\;\cdot \;m_{1}$ , where ‘ · ’ means the element-wise multiplication. Since the binary mask cannot be learned with backward propagation, Piggyback introduces a soft network $m_{1}^{r}={\left[m^{r}\right]}_{1,ij}$ . Each time, the algorithm selects a binary mask from the soft network by a threshold and copies the gradient of the binary mask to the soft network, as shown below:

(1) $$m_{1,ij}=\left\{\begin{array}{c} 1,\mathrm{if}\;ij\in \mathrm{topk}m^{r}\\ 0,\mathrm{otherwise} \end{array}\right.,\;\delta m_{1,ij}^{r}=\delta m_{1,ij}.$$

Then, for the following continual learning tasks, suppose we have learned task n−1 ; next, we will learn task *n*. In the experiment, to control the capacity of the network, we choose the threshold *k* by a ratio of the size of the network, i.e., $k=c∥W∥,c\in \left[0,1\right]$ .

However, the traditional Piggyback algorithm performs poorly when controlling quadruped robots. Despite high payoffs during training, test performance is poor. Even when the size of the base network is increased by a factor of $\frac{1}{c}$ , the performance of the piggyback network still fails to match that of regular dense networks. We believe that one reason for this is the choice of metrics. Freely chosen parameters have validity problems. That is, if none of a neuron’s output parameters are selected, its input parameters cannot be learned. Similarly, if too many of a neuron’s output parameters are selected, the importance of its input parameters will also increase. Therefore, we use an element-based ratio to select parameters, i.e., we ensure the same ratio of activated parameters for each neuron. This allows our network to achieve performance commensurate with its capacity. When the size of the basic network is increased, each parameter of the network can still operate normally, thus improving the learning ability of our network. So, the element-based ratio mask should be optimized:

(2) $$m_{1,ij}=\left\{\begin{array}{c} 1,\mathrm{if}\;ij\in \mathrm{topk}m_{i}^{r}\\ 0,\mathrm{otherwise} \end{array}\right.,\;\delta m_{1,ij}^{r}=\delta m_{1,ij}.$$

Selecting and learning a new part of W from scratch will be a naive way to learn subsequent tasks like Piggybacking. However, unlike the traditional continual learning hypothesis, tasks in quadruped robot control are much more general. Instead of learning completely new parameters, we perform further training on the originally learned network. Thus, we add some vacant parameters to the original parameters of task n−1 as the network of the new task *n*.

Following the idea of soft network from the WSN method [8], we further allow the binary masks m for different tasks to overlap, but only one parameter that is never selected by any task (vacant parameters) will be updated and one parameter that is selected by the previous task will not be updated, called the occupied parameters:

(3) $$\delta m_{n,ij}^{r}=\left\{\begin{array}{c} \delta m_{n,ij},\mathrm{if}\;m_{n,ij}^{\prime }=0\\ 0,\mathrm{otherwise} \end{array}\right.,$$

where $m_{n,ij}^{\prime }=\prod_{n^{\prime }\in \left\{1,\cdots ,n-1\right\}}m_{n^{\prime },ij}=0$ is a parameter is vacant ( =0 ) or occupied ( =1 ). W , m and $m^{r}$ are learned together. However, in the vanilla WSN method [8], the soft network is updated by a Straight-through Estimator [9], which needs to update the soft network while ignoring the mask. However, exploration and learning without the mask will be dangerous and inefficient for the robot. Therefore, unlike WSN, optimizing the soft network by Piggyback will suffer from low plasticity because the soft network cannot distinguish between free and occupied parameters. Once the selected parameters consist mainly of occupied parameters, the algorithm will stop learning. Therefore, for each new task *n*, we force at least λ vacant parameters to be introduced along with 1−λ occupied parameters to learn the new task *n*. Both vacant and filled parameters are selected by the soft network, i.e.,

(4) $$m_{n,ij}=\left\{\begin{array}{c} 1,\mathrm{if}\;m_{n,ij}^{r}\geq r_{\mathrm{vacant}}\;\mathrm{and}\;m^{*}\\ 1,\mathrm{if}\;m_{n,ij}^{r}\geq r_{\mathrm{occupied}}\;\mathrm{and}\;m^{*}\\ 0,\mathrm{otherwise} \end{array}\right.,\;\delta m_{1,ij}^{r}=\delta m_{1,ij}.$$

In this way, we can control the network parameters consumed by each task, avoiding the failure of some tasks during the learning process. In our experience, we choose the λ by proportion, which allows us to better control the learning capacity of the network.

After learning a task, only the binary mask will be saved.

### 2.4. Loss of Plasticity and Re-Initialization

Simply applying dynamic structure algorithms directly to the robot RL performs poorly, as shown in our experiments. The additional attached loss function and the loss of plasticity problem introduced by the dynamic structure make it difficult to learn tasks other than the initial task. We believe that this problem could possibly be solved by carefully tuning the hyperparameters and designing the network architecture and learning processes. However, such an approach would require re-tuning the hyperparameters from scratch each time we face a new task. It is well-known that tuning hyperparameters for quadruped robot control problems is an extremely complex and tedious process [10]. This would cause our method to lose its plug-and-play capability.

In order to compensate for the loss of plasticity, we propose to reinitialize the base and soft networks after learning each task. Besides leaving the occupied parameters of the base network unchanged, other parameters are reset to random initialization states (our experiments show that re-initializing the soft network for vacant parameters or the entire soft network leads to similar results). Note that for vacant parameters, although they are not selected in the end, they may still have been updated during optimization. Meanwhile, random initialization of the soft network also avoids problems where soft network values become too large or too small during the previous task, preventing learning in the new task. Also, since we are optimizing the soft network using the Piggyback algorithm and using the gradient of m instead of $m^{r}$ , the size of $m^{r}$ does not affect the gradient scale. Thus, the convergence speed of the soft mesh is influenced by the scale of $m^{r}$ .

Another factor affecting plasticity is the exploration ability of the robot, which is correlated with the entropy of robot actions. Robots that output actions with higher entropy can retain higher levels of exploration in following environments. With the learned skills and knowledge, the robot will tend to choose the actions that is stable and secure in order to avoid degradation in performance. However, the stable skills that learned by past tasks cannot obtain the optimal performance in the new task without exploration with more flexible and random actions, so that to keep the entropy when selecting the occupied parameters is important. Therefore, we have introduced a reward function in our robot learning to encourage the robot to maintain the soft network with high entropy. Therefore, avoiding adding the entropy into the loss function of the base network, we take it as a term of the loss function of the soft network; that is:

(5) $$m_{n,ij}^{r}=\mathrm{Normal}\left(m_{n,ij}^{r,\mathrm{base}},\sigma^{2}\right),\;\delta m_{n,ij}^{r}=\left\{\begin{array}{c} \delta m_{n,ij}+\delta H,\mathrm{if}\;m_{n,ij}^{\prime }=0\\ 0,\mathrm{otherwise} \end{array}\right.,$$

where $H=\frac{1}{2}\ln\left(2\pi e\sigma^{2}\right)$ is the entropy, $m_{n,ij}^{r,\mathrm{base}}$ is the mean of the distribution of the soft network, and σ is the variance of the output of the soft network. After learning several steps (100 in our experiment), we reduce the entropy on the soft network and let the base network enhance the final rewards.

Forward transferability and maintaining plasticity are a trade-off in continual learning. Our algorithm reconciles this dilemma well by reinitializing vacant parameters while still allowing access to occupied parameters.

### 2.5. Sim to Real in Continual Learning

Another aspect of the real-world robot control problem is the sim-to-real shift. Although they have learned in simulation environments, quadruped robots still need to be fine-tuned in real environments in order to be deployed there. However, due to conditional constraints, it is ideal but often infeasible to immediately fine-tune in the real environment after learning a task in simulation. Thus, fine-tuning a task in the real environment can still lead to catastrophic forgetting of other tasks. Therefore, we propose a method to isolate some parameters as private parameters for each task, which are not accessible by other tasks, to prevent the network from forgetting even after fine-tuning. Only these parameters are optimized during the real fine-tuning. In addition, our method allows for multiple learning of the same task to adapt to small variations in the environment or task. Due to the complexity of control tasks, robots often find it difficult to fully explore the state space once. Repeated learning of the same task is important in reinforcement learning [11]. In practice, we directly use the soft network to select parameters. The soft mesh cannot accurately reflect the importance of the parameters, but has a lower computational cost.

### 2.6. Compare to the Past Works

Our work is mainly based on the two algorithms, Piggyback [7] and Winning Soft-network [8]. We would like to emphasize the difference between our method and these two methods here. Comparing with Piggyback, our method can leverage experience from past tasks. This not only improves learning capability for subsequent tasks, but also saves space usage for the subsequent tasks. Also, to address the issue of loss of plasticity in Piggyback, we employed re-initialization and maintaining the entropy of soft-network to increase the plasticity, ensuring that our algorithm can extensively explore all parameters. Then, comparing with the WSN, our method uses the Piggyback soft-network updating, which not only can be easily used in RL setting, but also saves the computation time. Finally, we introduce a simple way to efficiently choose and keep some private parameters for sim-to-real transfer.

## 3. Results

### 3.1. Compared Methods

To evaluate the performance of our method, we compare it with six widely used continual learning methods:

- Experience Replay (ER) [12]: a basic rehearsal-based continual method that adds a behavior cloning term to the loss function of the policy network.
- Averaged gradient episodic memory (AGEM) [13]: a method based on gradient episodic memory that uses only a batch of gradients to limit the policy update.
- Elastic weight consolidation (EWC) [14]: constraining changes to critical parameters by the Fisher information matrix.
- Riemannian Walk (R-Walk) [15]: a method adds a parameter importance score on the Riemannian manifold based on EWC.
- Synaptic Intelligence (SI) [16]: a method that constrain the changes after each optimization step.
- Packnet [17]: a method to sequentially “pack” multiple tasks into a single network by performing iterative pruning and network re-training.

We evaluate these methods using the same network architecture as our method, but a multi-head network. For the two rehearsal-based methods compared (ER and AGEM), we used a replay buffer with a capacity of a trajectory of the data collected for each task, which is a large proportion for general applications [18]. For each task, we select 20% of the parameters and keep 2% of the parameters for online tuning.

### 3.2. Implementation Details

Each task in the sequence is a robot locomotion task, which we model as an RL task. According to [19], we define a Q-function $Q\left(s_{t},a_{t}\right)$ , a policy $\pi \left(a_{t}|o_{t},z_{t},v_{t}\right)$ and a context-aided estimator network (CENet) $C\left({\tilde{o}}_{t},v_{t}|o_{t}^{H}\right)$ . Q-learning methods train a Q-function by iteratively applying the Bellman operator $B^{*}Q\left(s_{t},a_{t}\right)=r\left(s_{t},a_{t}\right)+\gamma E_{s_{t+1}∼P\left(s_{t}|s_{t},s_{t}\right)}\left(\max_{a_{t+1}}\right.$ $\left.Q\left(s_{t+1},a_{t+1}\right)\right)$ . By introducing the privileged observation $s_{t}$ only into the Q-network, the agent (policy) also can makes decisions without privileged observation when evaluating in the real world. The implementation also follows the definition of the privileged observation $s_{t}$ in [19]. The CENet from [19] is used to jointly learn to estimate and infer a latent representation of the environment. The architecture of the CENet consists of a single encoder and a multi-head decoder to encode $o_{t}^{H}$ into $v_{t}$ and ${\tilde{o}}_{t}$ .

Our basic actor-critical RL method is from [19]. For details on the specific state space S , action space A , and reward function *r*, we follow [19]. We also list the elements of the reward function in Table 1, where $\cdot {\;}_{target}$ indicates the desired values. *x*, *y*, and *z* are defined on the body frame of the robot, with *x* and *z* pointing forward and upward, respectively. *g*, $v_{z}$ , ω , *h*, *p*, and $v_{f}$ are the gravity vector projected onto the robot’s body frame, linear velocities in the z-plane, yaw rate, body height above ground, foot height, and foot lateral velocity, respectively.

We mainly follow the setting of the RL network in [19]. Both the actor and the critic are multi-layer perceptron (MLP) with three hidden layers of 512, 256, and 128 neurons, respectively, each with ReLU non-linearity. The CENet is also a multi-layer perceptron with two hidden layers of 256 neurons for the encoder and decoder. We also use Adam [20] with a learning rate of 0.001 to update both the basic network and the soft network of actor, critic, and CENet. According to [21], we increase the epsilon of Adam to 1 × 10^−5^. We update each task by 500 epochs. Unlike the vanilla Piggyback algorithm, we initialize the soft network with a random Gaussian noise, which works better than a fixed value for all tasks except the first.

For each task, we keep 20% private parameters and keep 2% parameters for sim-to-real learning. In the 20% private parameters, we choose 25% vacant parameters and 75% occupied parameters so that the λ is 25%. For each task, we train it 500 epochs and turn to the next task.

### 3.3. Experimental Environments

**Figure 3:**
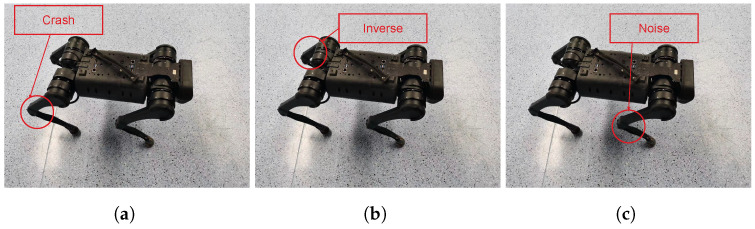
(**a**) In the Leg Crash task, we set the output of each leg into zero, respectively. (**b**) In the Leg Inversion task, we inverse the output of each leg, respectively. (**c**) In the Leg Noise task, we add a random noise into the output of each leg, respectively.

We will discuss the three sets of tasks we are evaluating (see Figure 3).

- Leg Crash: we set the output of different legs of the robot to zero for each task to simulate the situation where the leg crashes and cannot move.
- Leg Inversion: for each task, we invert the outputs of the neural network for one leg of the robot.
- Leg Noise: we add random Gaussian noise to the output of the robot. From the first task to the fourth task, we add the noise to the left front leg, the right front leg, the left rear leg, and the right rear leg.

In order to make our experiments as long as possible and increase the number of tasks, we randomly mix the three cases to obtain the final experimental tasks.

## 4. Discussion

### 4.1. Episode Reward

**Figure 4:**
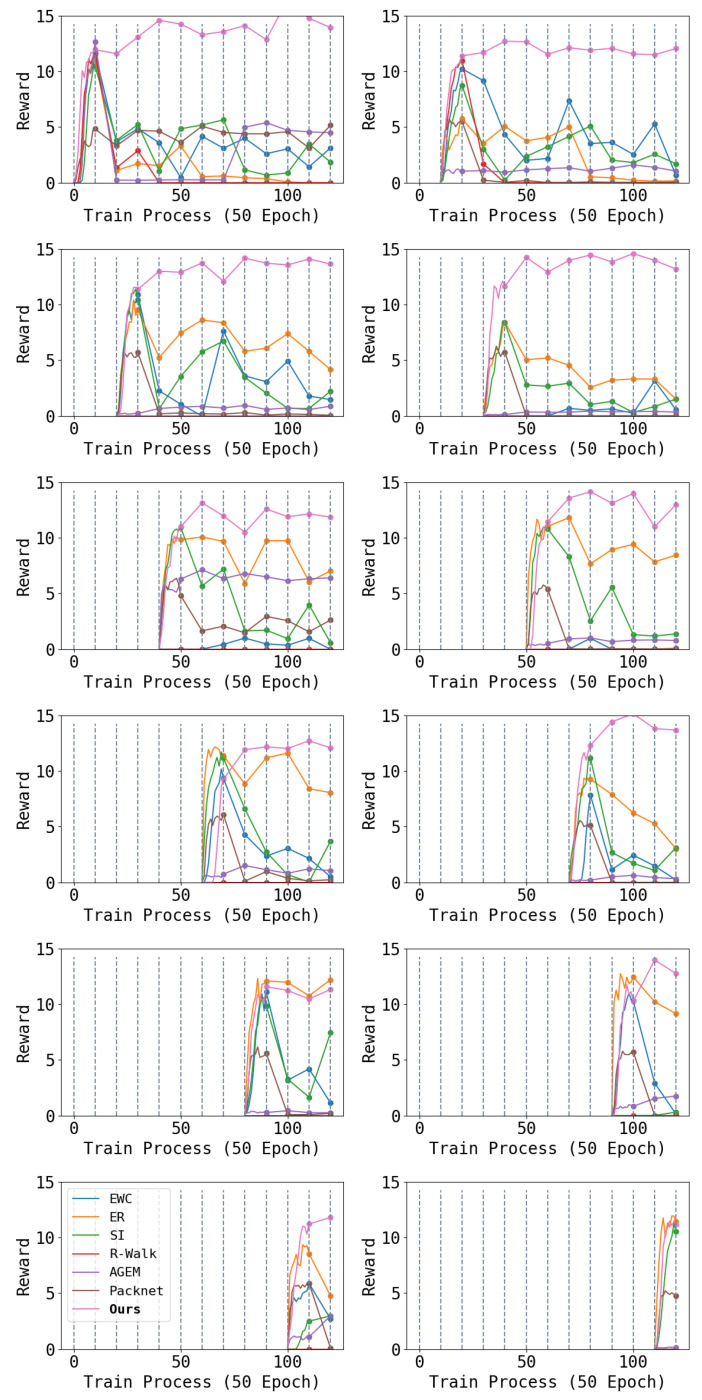
Each figure shows the performance of one task during the entire learning phase: the first row is for tasks 1–2, the second row is for tasks 3–4, and so on. Throughout the learning process, we train each task 500 epochs and switch to the next task. The first 500 epochs of each task show the reward during training, while subsequent data are results of testing the performance on that task each 500 epochs. Higher results are better. We can see that our algorithm maintains the performance achieved during training on all tasks, while all baselines exhibit decreased performance during later testing.

We report part of the result in Figure 4. From the figure, we can see that a traditional continual learning architecture cannot solve the complex problem of the locomotion of a quadruped robot. Among them, regularization-based algorithms (EWC, SI, and R-Walk) have difficulty maintaining acquired performance. After several tasks, the performance of learned tasks decreases notably. In comparison, rehearsal-based algorithms (ER and AGEM) perform better, but robot performance still declines after multiple tasks. This may be because robot locomotion poses higher requirements on network parameter stability. Even small changes in network parameters can lead to a damaged locomotion ability. The pruning-based dynamic-architecture-method (Packnet) performs the worst because the additional L1 loss will affect the learning of the robot.

**Figure 5:**
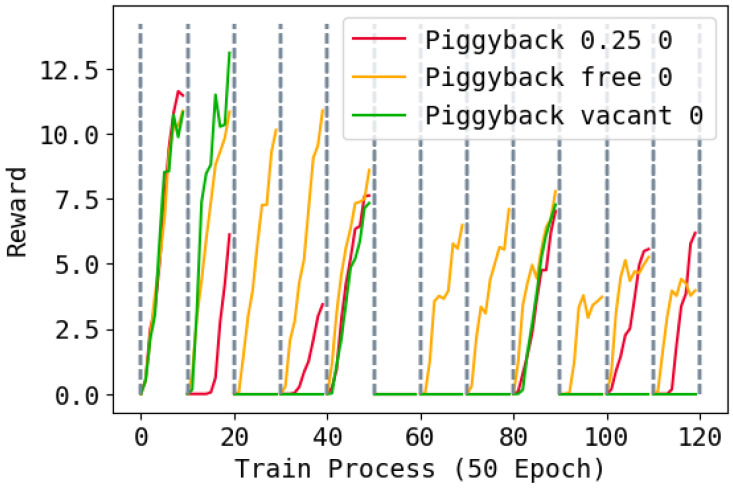
Training rewards of the Piggyback method during the whole training process. A value of 0.25 means each task uses 25% vacant parameters and 75% occupied parameters; free means select using vacant parameters and the occupied parameters freely like WSN; vacant means only use the vacant parameters for new tasks. The task changes every 500 epochs. Higher is better.

Finally, we report the performance of the Piggyback methods with different hyper-parameters and random seeds in Figure 5. It can be seen that Piggyback perform well in first few tasks, but the learning ability will reduce after learning several tasks. This is because the Piggyback method cannot leverage the occupied parameters as well as ours.

In comparison, our method not only has significant advantages in mitigating catastrophic forgetting, but also maintains better plasticity as the number of learned tasks increases than baselines. Although each task requires a long exploration and training process, our method can still maintain the original locomotion ability. Even though we use only 20% of the parameters for a task, the learning ability is sufficient to learn such complex RL tasks as controlling quadruped robots without enlarging the neural network. Furthermore, even though our method has slightly lower performance for the first task in some cases compared to the incomplete version, the higher plasticity will be reflected in the performance of subsequent tasks. The learning capabilities of AGEM and R-Walk significantly decrease after learning several tasks, showing an inability to learn new tasks to adequate levels during training. In contrast, EWC, ER, and SI ensure higher learning ability on new tasks, and Piggyback suffers almost no loss of plasticity problem.

### 4.2. Commands Tracking

**Figure 6:**
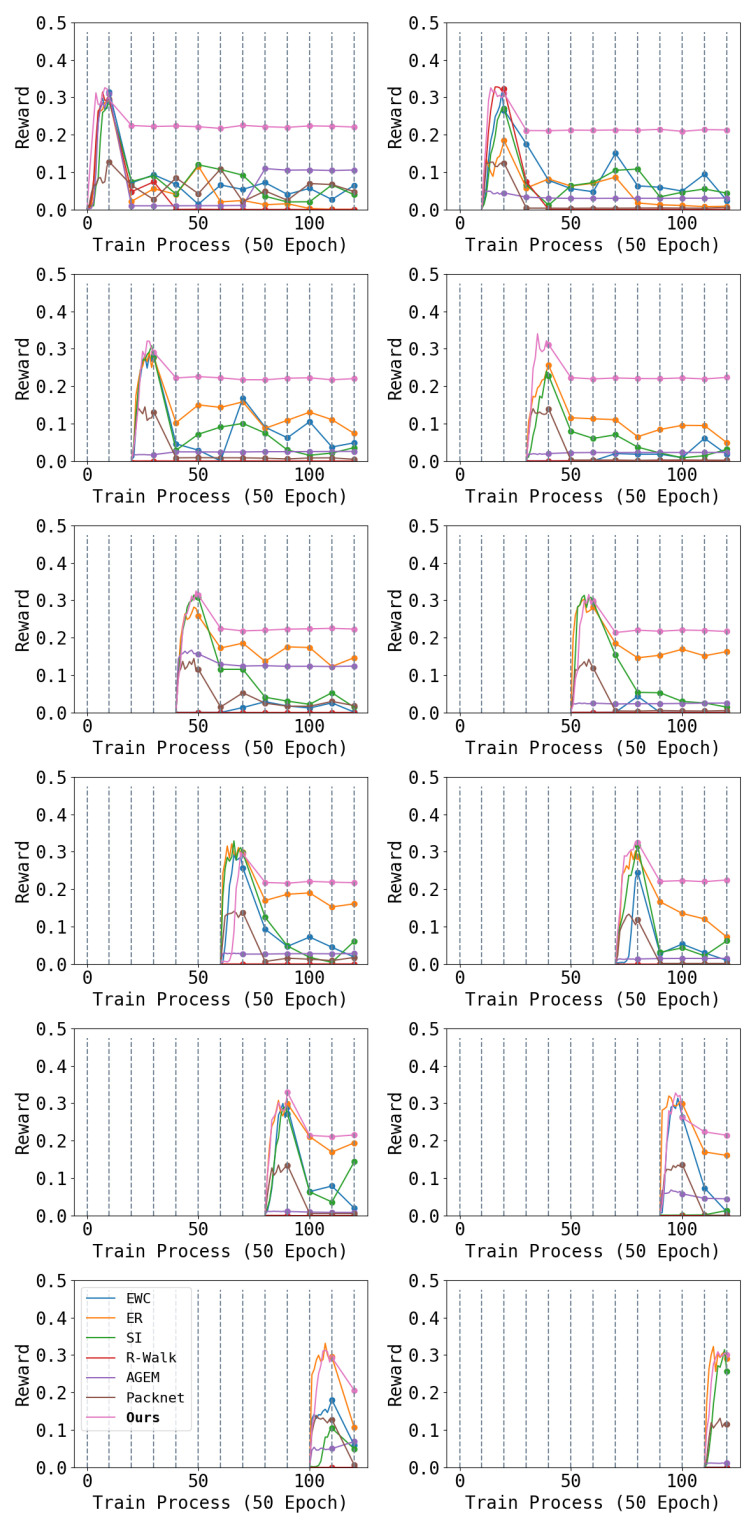
Each figure shows the performance of tracking the line velocity of one task during the entire learning phase: the first row is for tasks 1–2, the second row is for tasks 3–4, and so on. Throughout the learning process, we switch the robot to another task every 500 epochs. The first 500 epochs of each task show the performance of tracking the line velocity during training, while subsequent data are the results of testing the performance on that task for each 500 epochs. Higher results are better. We can see that the performance of our method only drops just after training (because of the shift between training and testing), and can keep a steady performance in the remaining learning process.

**Figure 7:**
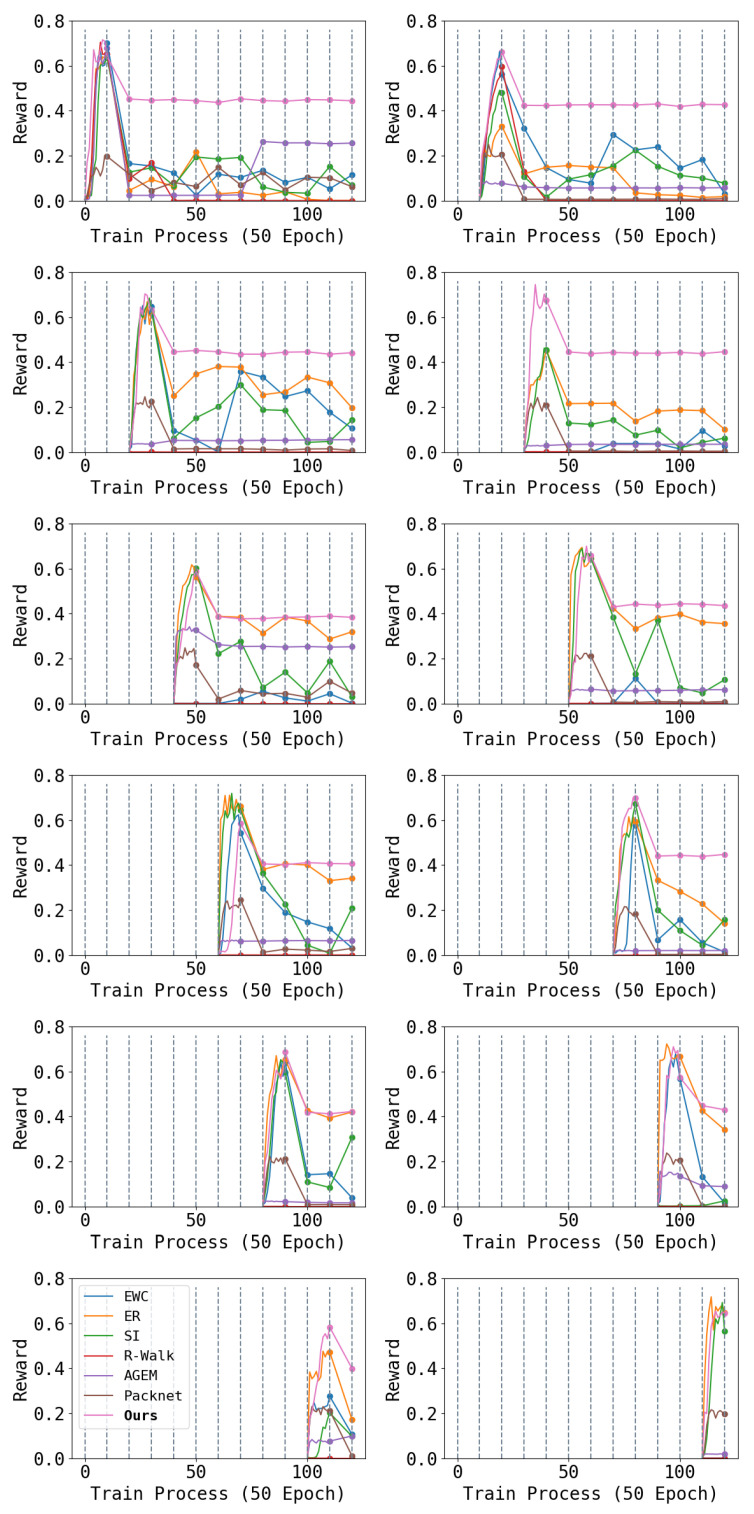
Each figure shows the performance of tracking the angle velocity of one task during the entire learning phase: the first row is for tasks 1–2, the second row is for tasks 3–4, and so on. Throughout the learning process, we switch the robot to another task every 500 epochs. The first 500 epochs of each task show the performance of tracking the line velocity during training, while subsequent data are the results of testing the performance on that task for each 500 epochs. Higher results are better. We can see that the performance of our method only drops just after training (because of the shift between training and testing), and can keep a steady performance in the remaining learning process.

The other two important arguments for robot locomotion are the line velocity and the angle velocity. These two arguments are commonly used to measure the correct locomotion of the quadruped robots [19]. We compare the difference between the output velocity and input command with three random commands. Each command is the expected line speed sampled from $\left[-1.0,1.0\right]$ m/s and the angle speed sampled from $\left[-\frac{\pi }{2},\frac{\pi }{2}\right]$ rad/s. We give the tracking performance of the line velocity in Figure 6 and the angle velocity in Figure 7.

We can see that after learning the follow-up task, the ability to follow the moving commands decreases. In particular, the robot tends to reduce speed to ensure safety.

### 4.3. Effect of Re-Initialization and Entropy

**Figure 8:**
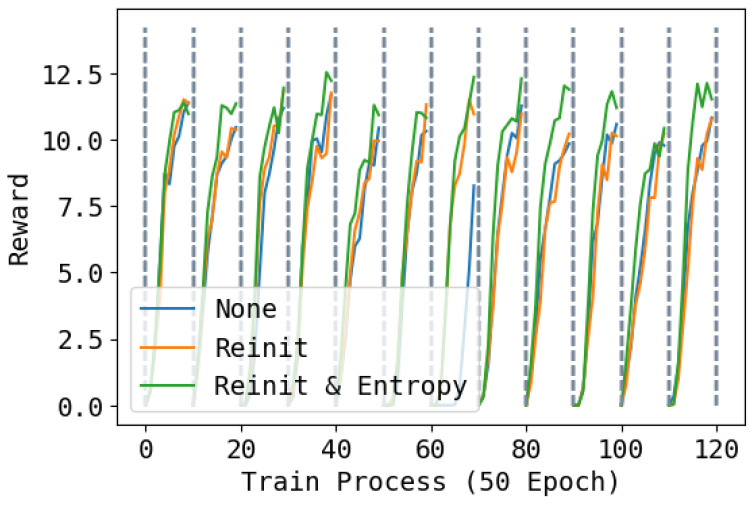
Training rewards during the whole training process. None means not to reinitialize; Reinit means to reinitialize unused parameters of the base network and the soft network; Reinit & Entropy means to reinitialize the unused parameters of the base networks and the soft network, and raise the entropy of the action when learning the soft network. The task changes every 500 epochs. Higher is better.

**Figure 9:**
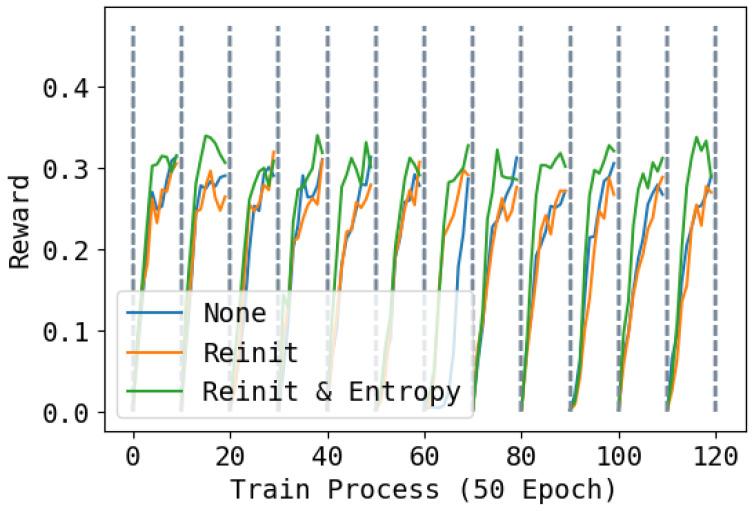
Training performance of tracking line velocity during the whole training process. None means not to reinitialize; Reinit means to reinitialize unused parameters of the base network and the soft network; Reinit & Entropy means to reinitialize the unused parameters of the base networks and the soft network, and raise the entropy of the action when learning the soft network. The task changes every 500 epochs. Higher is better.

**Figure 10:**
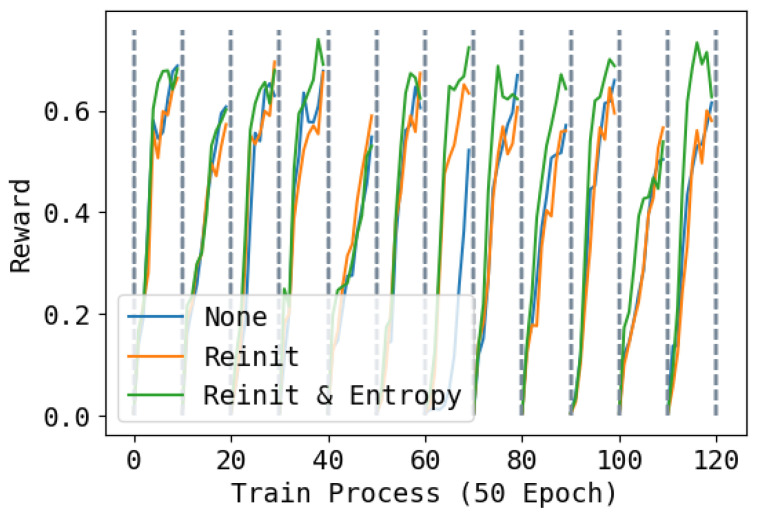
Training performance of tracking angle velocity during the whole training process. None means not to reinitialize; Reinit means to reinitialize unused parameters of the base network and the soft network; Reinit & Entropy means to reinitialize the unused parameters of the base networks and the soft network, and raise the entropy of the action when learning the soft network. The task changes every 500 epochs. Higher is better.

In Figure 8, Figure 9 and Figure 10, we show the reward during training on each task under three different re-initialization schemes. We can see that re-initialization enhances the plasticity for the following tasks. For most tasks, whether the base network is reinitialized also does not have an obvious impact, but for some tasks, not re-initializing parameters can lead to slower training. Also, optimizing the entropy for soft network also hastens the learning process of the following tasks.

### 4.4. Forward Transfer

**Figure 11:**
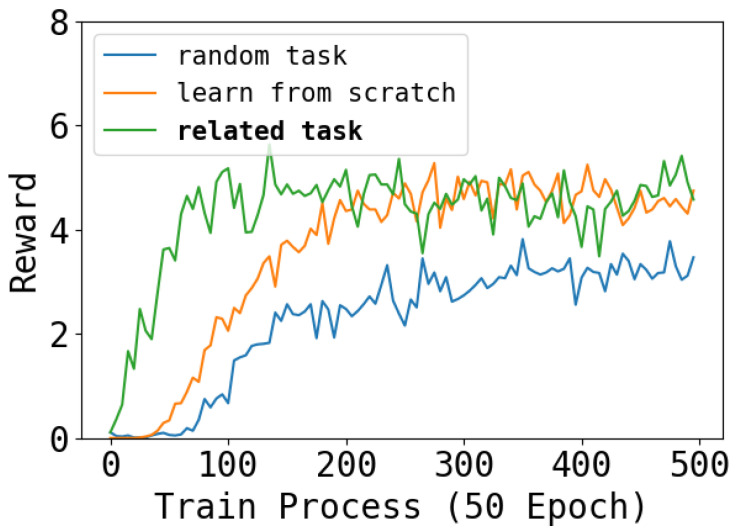
The learning process of the mixed environment. We can see that learning a new task that combination of two learned tasks from a combination of the soft network can speed up the training process.

An important capability of our architecture is forward transfer. We evaluate the forward transfer ability by a newly designed environment consisting of all the interference of tasks. We mix a new task by two learned task, i.e., a robot have both a inverse right back leg and a noise right forward leg. Then, we learn this new task with a soft network that the chosen parameters by the task “inverse right back leg” and the task “noise right forward leg” has a higher initialization than other occupied parameters. We called this way “related task”. As a contrast, we propose two other learning ways: “random select”, which initialize the soft network by other two random tasks, and “learn from scratch”, which does not change the initialize way. The result is shown in Figure 11. This result reflects the forward transferability of our methods. We can see that the “related task” learns faster than “learn from scratch” because it has a better foundation. Then, “random task” learn the slowest and cannot match the performance because the extraneous parameters interfere with the learning process.

## 5. Conclusions

In this paper, we propose an algorithm that performs continual learning on a quadruped robot control using a dynamic architecture approach. We point out that complex quadruped control environments make traditional continual learning algorithms difficult to adapt, while dynamic-architecture-based continual learning algorithms can achieve greater advantages with their excellent stability. We also propose to maintain the plasticity of the policies by resetting the parameters to avoid plasticity loss, which has a significant impact on the final performance. Finally, our experiments validate that our algorithm can enable positive transfer for skill composition, demonstrating the unique advantages of continual learning algorithms.

## Figures and Tables

**Table 1:** Reward structure.

Term	Equation	Weight
linear velocity tracking	$\exp\{-|v_{xy}-v_{xy}^{cmd}{|}^{2}/0.25\}$	1.0
angular velocity tracking	$\exp\{-|\omega_{z}-\omega_{z}^{cmd}{|}^{2}/0.25\}$	0.5
z velocity	$v_{z}^{2}$	−2.0
roll-pitch velocity	$|\omega_{xy}{|}^{2}$	−0.05
orientation	${\left|g\right|}^{2}$	−5.0
joint limit violation	$\sum_{j=1}^{12}\phi \left(q_{bj},q_{bj,lower},q_{bj,upper}\right)$	−10.0
joint torques	${\left|\tau \right|}^{2}$	$-2\times 10^{-4}$
joint accelerations	$|{\ddot{q}}_{b}{|}^{2}$	$-2.5\times 10^{-7}$
body height	${\left(h-h_{target}\right)}^{2}$	−30.0
feet clearance	${\left(p-p_{target}\right)}^{2}*v_{f}$	1.0
thigh/calf collision	$1_{collision}$	−1.0
action smoothing	$|a_{t-1}-a_{t}{|}^{2}$	−0.01

## Data Availability

No new data were created or analyzed in this study. Data sharing is not applicable to this article.
